# Binocular pattern deprivation interferes with the expression of proteins involved in primary visual cortex maturation in the cat

**DOI:** 10.1186/s13041-015-0137-7

**Published:** 2015-08-14

**Authors:** Karolina Laskowska-Macios, Julie Nys, Tjing-Tjing Hu, Monika Zapasnik, Anke Van der Perren, Malgorzata Kossut, Kalina Burnat, Lutgarde Arckens

**Affiliations:** Laboratory of Neuroplasticity, Nencki Institute of Experimental Biology, 02-093 Warsaw, Poland; Laboratory of Neuroplasticity and Neuroproteomics, KU Leuven - University of Leuven, 3000 Leuven, Belgium; Laboratory for Neurobiology and Gene Therapy, KU Leuven - University of Leuven, 3000 Leuven, Belgium

## Abstract

**Background:**

Binocular pattern deprivation from eye opening (early BD) delays the maturation of the primary visual cortex. This delay is more pronounced for the peripheral than the central visual field representation within area 17, particularly between the age of 2 and 4 months [Laskowska-Macios, Cereb Cortex, 2014].

**Results:**

In this study, we probed for related dynamic changes in the cortical proteome. We introduced age, cortical region and BD as principal variables in a 2-D DIGE screen of area 17. In this way we explored the potential of BD-related protein expression changes between central and peripheral area 17 of 2- and 4-month-old BD (2BD, 4BD) kittens as a valid parameter towards the identification of brain maturation-related molecular processes. Consistent with the maturation delay, distinct developmental protein expression changes observed for normal kittens were postponed by BD, especially in the peripheral region. These BD-induced proteomic changes suggest a negative regulation of neurite outgrowth, synaptic transmission and clathrin-mediated endocytosis, thereby implicating these processes in normal experience-induced visual cortex maturation. Verification of the expression of proteins from each of the biological processes via Western analysis disclosed that some of the transient proteomic changes correlate to the distinct behavioral outcome in adult life, depending on timing and duration of the BD period [Neuroscience 2013;255:99-109].

**Conclusions:**

Taken together, the plasticity potential to recover from BD, in relation to ensuing restoration of normal visual input, appears to rely on specific protein expression changes and cellular processes induced by the loss of pattern vision in early life.

**Electronic supplementary material:**

The online version of this article (doi:10.1186/s13041-015-0137-7) contains supplementary material, which is available to authorized users.

## Background

Previous investigations emphasizing the molecular development of primary visual cortex typically dealt with its central visual field representation, or did not take a distinction between the central and peripheral visual field representations into account. Age-dependent expression profiles of proteins involved in neurite outgrowth, energy metabolism, synaptic development and neurotransmission are described [1–7]. Yet, when considering ocular dominance plasticity or synapse formation the development of the peripheral visual field representation in cat area 17 is slower than that of the central visual field representation [8, 9–12] (reviewed in [11]). In area 17 of marmoset monkey, Bourne and colleagues [13] showed that neurofilament protein patterns correlate with the maturation state of a given neocortical brain region and that the peripheral visual field representation in area 17 also achieves such a mature pattern later than the central visual field representation does.

In cat, lack of patterned visual input has been shown to differentially affect the development of the X- and Y-dominated functional pathways carrying information predominantly derived from the central versus the peripheral visual field respectively. At the level of the retina, early binocular pattern deprivation (BD), an animal model of congenital cataract, if applied for 6 months, results in permanent changes in the number and dendritic tree stratification of Y-type motion-sensitive alpha retinal ganglion cells [14]. At the level of the thalamus, in the LGN, BD specifically affects the development of the Y-cell pathway, processing motion-sensitive visual information, but not the X-cell pathway, dominant in processing high resolution visual information (for review see [15]). Importantly, the parallel motion perception impairments observed in cat have also been described in patients with congenital binocular cataract (cat: [16, 17, 18]; human: [19, 20]). Furthermore, at the level of the visual cortex, early BD delays the maturation of peripheral area 17 to a larger extent than its central counterpart, as visualized by age- and cortical region-dependent expression changes for the activity reporter gene *zif268* in the visual cortex of BD kittens [8].

We therefore decided to apply a functional proteomics approach to find molecular correlates for the centro-peripheral developmental gradient in area 17 in order to identify important proteins underlying cortical-region specific maturation. To this end, we separately assessed the central and peripheral region of area 17 of 2- and 4-month old BD (2BD and 4BD; early onset BD) kittens and age-matched controls with normal visual experience. Age and the distinct BD-induced delay of cortical maturation were thus considered as factors influencing protein expression in relation to cortical maturation. Two-Dimensional Difference Gel Electrophoresis (2-D DIGE) combined with mass spectrometry and Ingenuity Pathway Analysis (IPA) allowed the prediction of relevant molecular pathways and biological processes. To validate these proteomics observations and to investigate and compare protein expression profiles in additional experimental conditions, Western analysis was also applied to homogenates from area 17 of 6-month old BD kittens, a late onset BD group binocularly deprived during the 3rd and 4th month of age after 2 months of normal vision (2N 2BD) and extra normally sighted controls of 1 and 6 months, and 2 years (Adult). Analysis of the developmental profiles of protein expression in the context of normal visual stimulation, early onset as well as late onset BD enabled us to determine if BD regulation of protein expression depended on time of onset of BD or not.

We demonstrate how specific developmental protein expression changes are postponed especially in the peripheral visual field representation under BD. In particular, early BD exerts an influence on protein expression in a direction suggestive of a negative regulation of neurite outgrowth, synaptic transmission and clathrin-mediated endocytosis.

## Results

### 2-D DIGE screening for protein expression changes related to cortical maturation

**Fig. 1:**
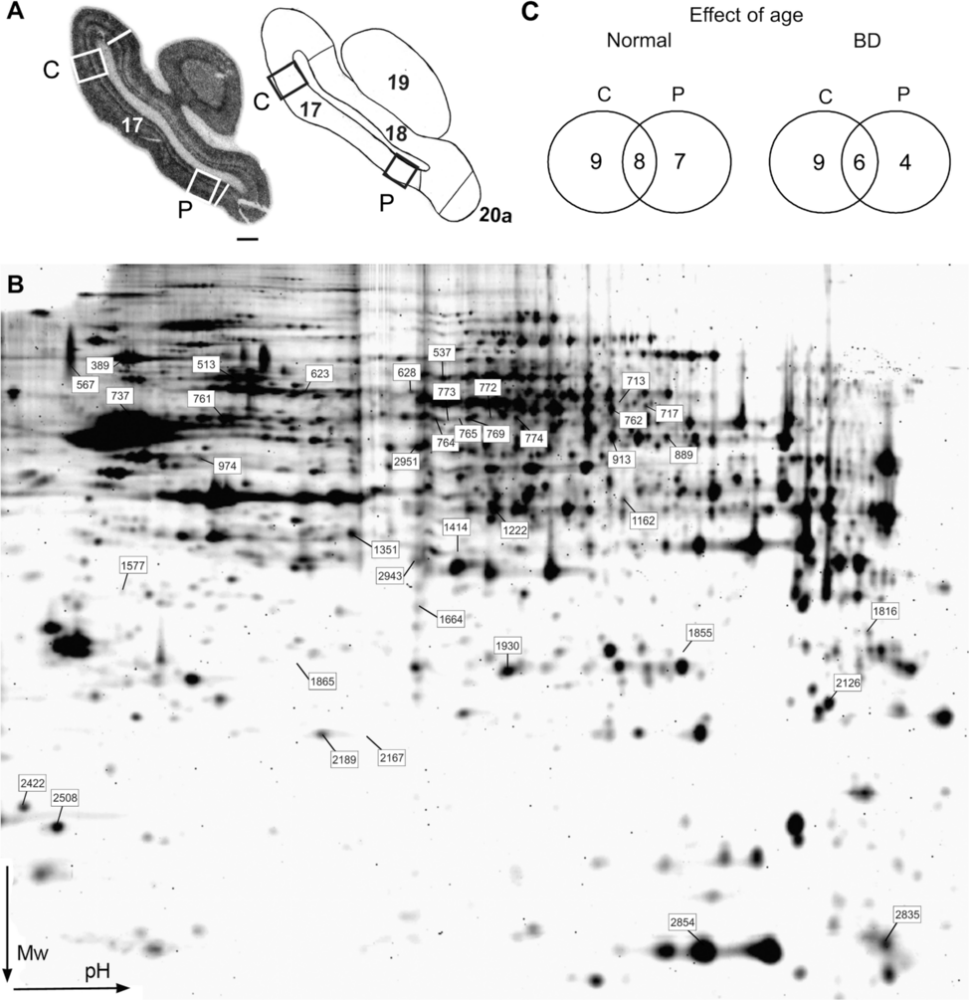
Tissue sampling for 2-D DIGE experiments. **a** Illustration of the tissue sampling in primary area 17: a frontal section of an *in situ* hybridization for *zif268* in area 17 of a 2-month old normal kitten and the relevant level of the Rosenquist retinotopic map (1985). We have collected brain tissue from the central (C) and peripheral (P) visual field representation (White/black boxes) at Horsley-Clarke coordinate posterior 7 (P7.0). White/black lines demarcate the areal borders of area 17. Scale bar: 1 mm. **b** Visualization of all differential spots on an image of a preparative 2-D gel. Spot numbers match with the information in Table 1. Mw: molecular weight. **c** Venn diagrams illustrating the number of age-related differential spots as a function of cortical region (central or peripheral area 17) for normal and BD cats

Age, BD, and centro-peripheral expression differences were considered valid parameters to chart molecular events in relation to cortical maturation (Fig. 1a-c, Table 1).

**Table 1:**
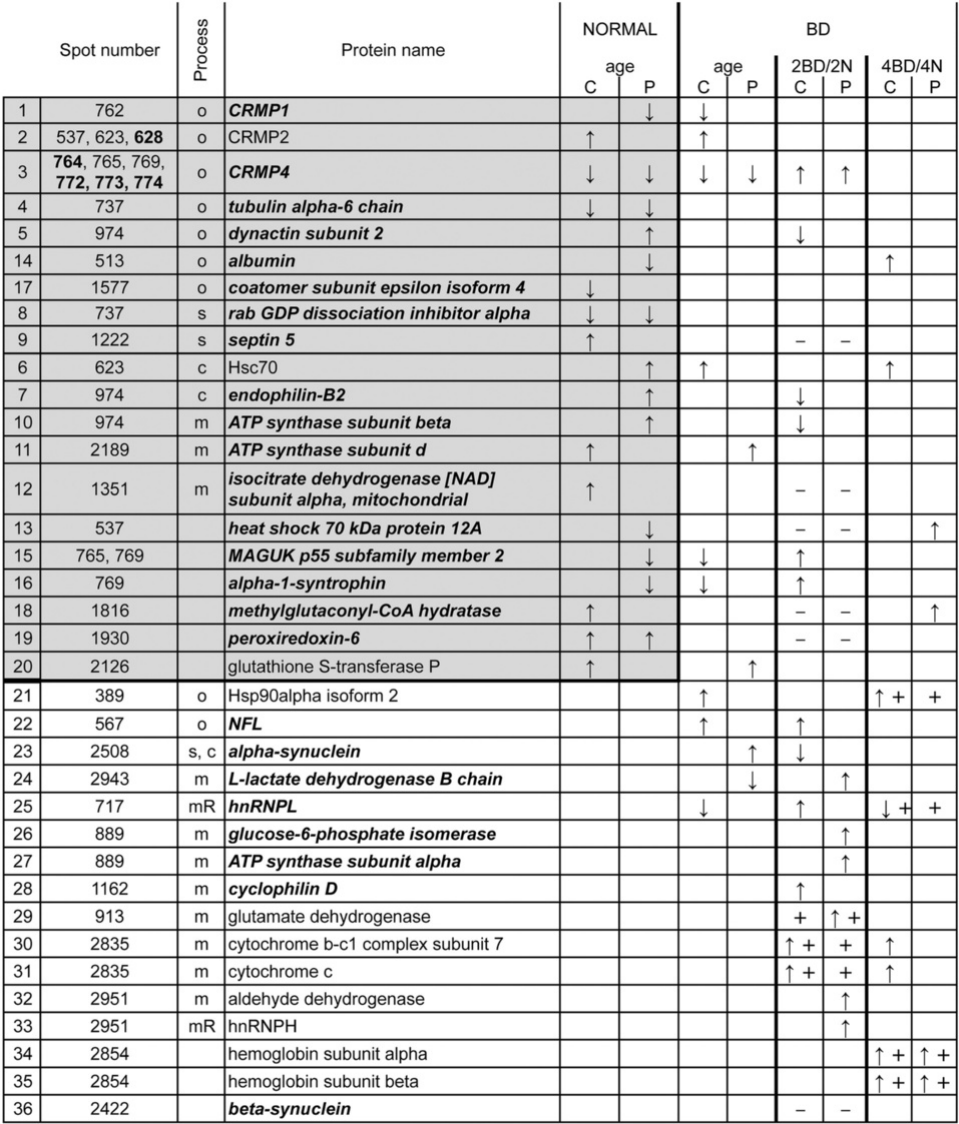
List of 36 differentially expressed proteins in area 17

Categorization was done according to the effect of age, BD, cortical region and the process that a particular protein is involved in. The first two ‘age-regulated’ columns visualize the presence and direction of difference (up or down) between the 2N and 4N group within central and peripheral area 17. The next two columns show the effect of age when reared under BD conditions, indicating the presence and direction of difference between the 2BD and 4BD group. In the next four columns, proteins were classified as ‘2BD-regulated’ or ‘4BD-regulated’ when their expression differed between a particular BD group and its age-matched normal control, or when there was a difference between the central and peripheral region in a BD group but not in its age-matched normal control group (+) or *vice versa* (−)*.* Between 4BD and 2N kittens, the peripheral region was molecularly similar for 97% (except protein in row 18), the central region was similar for 69% (25 proteins, marked in bold italics) and all other proteins showed higher expression in 4BD as compared with 2N. For CRMP2 and CRMP4 spot numbers that do not contain other proteins are in bold. Abbreviations: C: central; P: peripheral; o: outgrowth; s: synaptic transmission; c: clathrin-mediated endocytosis; m: energy metabolism; mR: mRNA metabolism and transport; ↑ – upregulation, or ↓– downregulation. All presented differences are significant, *p* < 0.05

### Analysis at spot level

Analysis of all the spot expression patterns revealed a total of 39 differentially expressed spots (Fig. 1b). When visualizing the effect of age on the number of differential spots for central and peripheral area 17 for normal and BD animals in a Venn diagram (Fig. 1c), a first indication for a reduced effect of age on peripheral area 17 becomes apparent with only 4 differential spots for peripheral area 17 of BD cats versus 7 for normal subjects.

### Analysis at protein level

Table 1 summarizes the results of a similar analysis upon mass spectrometric identification of the 36 proteins in 32 spots (see Material and Methods, Additional file 1: Table S1).

#### Normal

Comparison of the protein expression patterns within the central or the peripheral regions in area 17 between kittens of 2 and 4 months revealed an age-dependent expression level for 20 proteins (grey box). Only four proteins (rows 3, 4, 8 and 19) were similarly regulated in the central and peripheral region, whereas seven proteins were specific for central and nine for peripheral area 17.

#### BD

When probing for the effect of early BD on these age-specific expression patterns, only for CRMP2 and CRMP4 the expression changed in the same direction for both regions (rows 2 and 3). Eighteen out of twenty proteins did not show the same age-dependent modulation as in normal kittens. Instead, the factor age identified five additional proteins in BD kittens, not differential between 2N and 4N (rows 21–25). Comparison of the BD-subjects with their age-matched normal controls (columns 2BD/2N and 4BD/4N) revealed an extra set of 11 proteins (rows 26–36) with an expression deviating from normal levels, resulting in a list of 36 differential proteins with a potential role in cortical maturation.

In general, age-dependent protein expression changes occurred less frequently in peripheral than in central area 17 under BD (five versus nine proteins; column BD/age) indicating that protein expression changes relevant to area 17 maturation may be postponed, especially in the peripheral region. Likewise, centro-peripheral differences in protein expression were observed only at 2 months in normal animals (proteins in rows 9, 12, 13 and 18 were upregulated in the peripheral region; 19 and 36 upregulated in the central region; Additional file 1: Table S1), whereas in BD animals proteins showed a centro-peripheral expression gradient for both age groups (three proteins in 2BD and four in 4BD; indicated by ‘+’ in columns 2BD/2N and 4BD/4N). Importantly, when comparing expression of all identified proteins between 4BD and younger normal controls (2N), for the peripheral region 97 % did not differ (all proteins except for methylglutaconyl-CoA hydratase, row 18) whereas for the central region, 69 % did not (column 3, proteins in bold and italic). Also most of the proteins with a different expression between the 2N and 4N groups did not change expression with age in BD subjects. Together, these findings are a strong indication that especially the peripheral region of area 17 still resembles that of younger normal controls due to a BD-induced delay in maturation.

### Classification of identified proteins according to biological function

To reveal molecular pathways and biological functions potentially involved in cortical maturation, IPA software was applied to categorize the data set of the 36 identified proteins. The first two essential canonical pathways were ‘*Parkinson’s Signaling*’, containing synuclein alpha, septin 5, cytochrome c (*p* = 5.7E-04); and ‘*Mitochondrial Dysfunction*’, including the proteins involved in energy production, such as ATP synthase subunit alpha and beta, cytochrome c and cytochrome b-c1 complex subunit 7 (*p* = 7.07E-04). A significant canonical pathway was also ‘*Semaphorin Signaling in neurons*’ regulating neurite outgrowth, where CRMP1, CRMP2 and CRMP4 are the main players (*p* = 4.25E-03). Another canonical pathway was ‘*Clathrin-mediated Endocytosis Signaling*’ involving Hsc70, endophilin-B2 and albumin (*p* = 9.56E-02). Albumin was also a member of ‘*Caveolar-mediated Endocytosis Signaling*’ canonical pathway, which involved coatomer subunit epsilon isoform 4 (*p* = 1.15E-01). In the nervous system, albumin is a cargo protein transported to astrocytes not via clathrin- but caveolar-mediated endocytosis to promote synthesis and release of the neurotrophic factor oleic acid and subsequent neuronal differentiation and outgrowth [21]. Albumin was therefore not considered as a protein involved in clathrin-mediated endocytosis, but as a molecule transported via caveolar-mediated endocytosis.

**Fig. 2:**
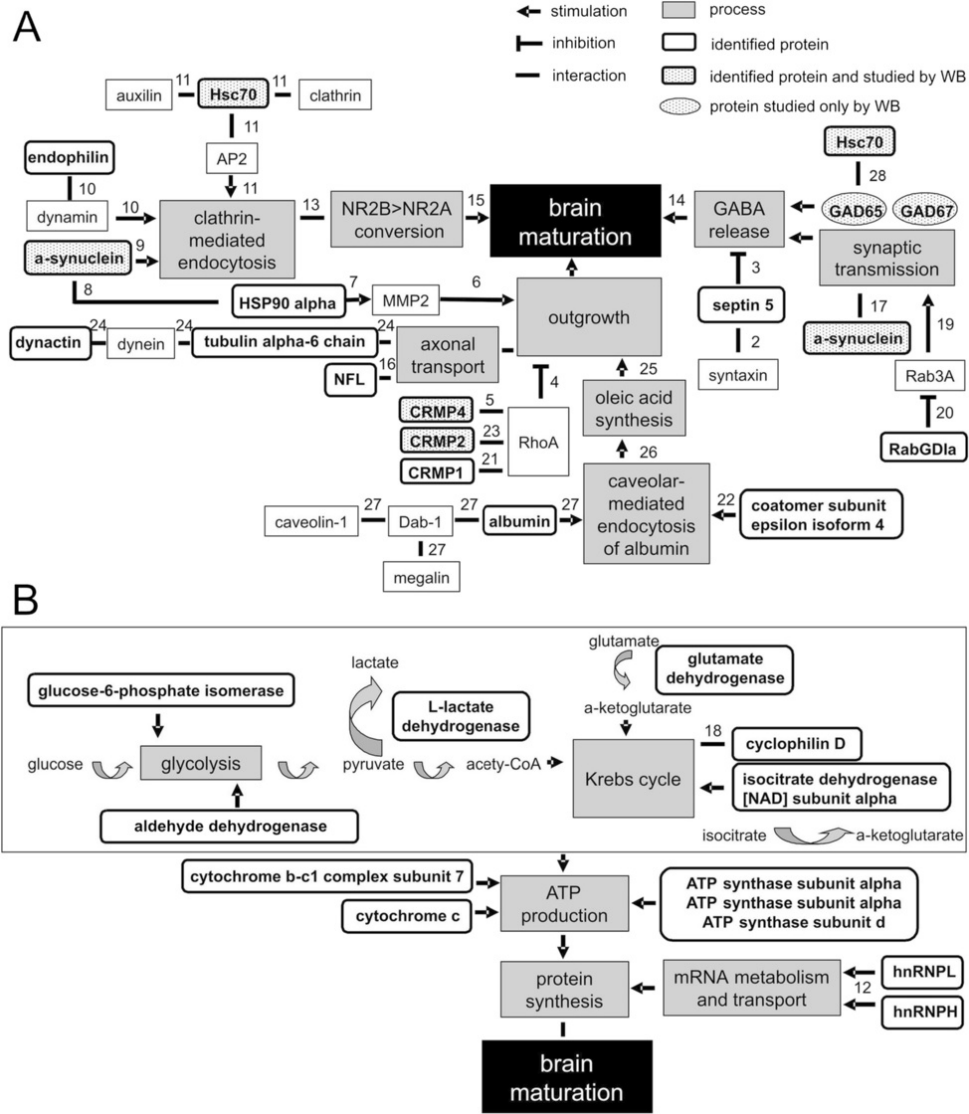
Proposed schematic overview of protein interactions. Interactions among detected proteins and proteins described in literature that are involved in processes with regard to the development of primary area 17: *outgrowth, synaptic transmission, clathrin-mediated endocytosis* (**a**) and *metabolism* (**b**). Interactions are accompanied by numbers, referring to corresponding references: **1.** [85] **2.** [86]; **3.** [87]; **4.** [88]; **5.** [49]; **6.** [89]; **7.** [90]; **8.** [91]; **9.** [72]; **10.** [92]; **11.** [93]; **12.** [94]; **13.** [69]; **14.** [95]; **15.** [7]; **16.** [96]; **17.** [32]; **18.** [97]; **19.** [98]; **20.** [99]; **21.** [100]; **22.** [101]; **23.** [102]; **24.** [103]; **25.** [104]; **26.** [105]; **27.** [21]; **28.** [25]. Grey rectangles – processes, rectangles with rounded corners – differential identified proteins; rectangles with rounded corners and dotted background – proteins studied by WB; proteins in an oval frame and dotted background – proteins studied by WB only; arrows – stimulation; line – interaction; perpendicular lines – inhibition

Based on the IPA output we plotted a scheme presenting interactions between the identified proteins and their involvement in different biological processes. Figure 2a shows the interactions between 27 proteins related to three biological processes: outgrowth, clathrin-mediated endocytosis and synaptic transmission. Figure 2b illustrates a separate pathway linking the 11 proteins important for energy production. As eight out of the eleven identified mitochondrial energy metabolism-associated proteins had a higher expression in the 2BD group as compared to age-matched normal controls (Table 1), their upregulation suggests a higher energy demand to support neuronal activity and thus corresponds well with the previously revealed hyperactivity in area 17 of 2BD subjects [8].

### The effect of age and BD on developmental profiles of proteins involved in outgrowth, synaptic transmission and clathrin-mediated endocytosis

To validate the 2-D DIGE results and IPA interpretation, we performed Western analysis for 4 proteins with a significant change in expression and belonging to the main biological processes highlighted in Fig. 2a: CRMP2 and CRMP4 for *outgrowth*, alpha-synuclein for *synaptic transmission* and Hsc70 for clathrin-mediated endocytosis. For *synaptic transmission* we additionally analyzed GAD65 and GAD67 expression in all experimental conditions as markers of inhibitory transmission.

#### Outgrowth

**Fig. 3:**
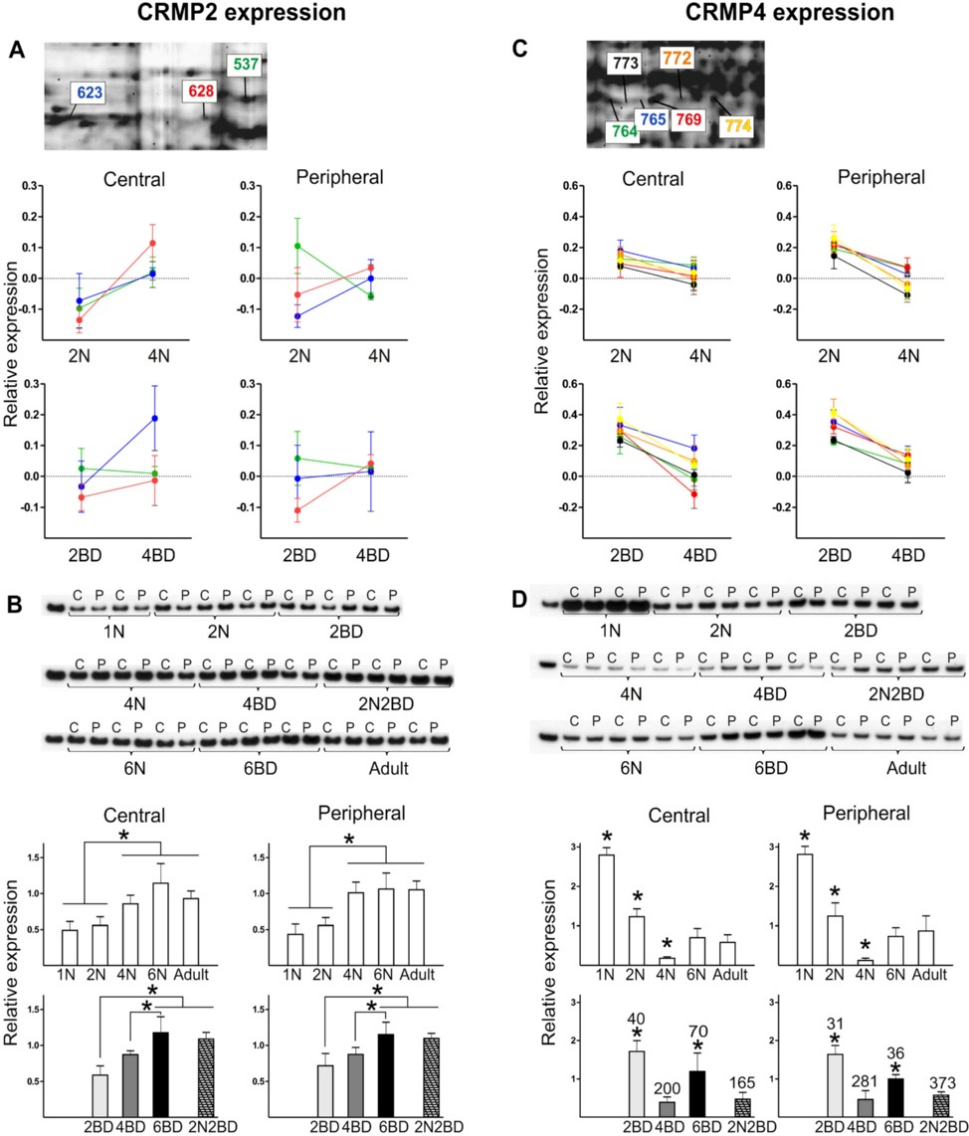
CRMP2 and CRMP4 protein expression analysis. Visualization and analysis of the relevant spots from the 2-D DIGE experiments containing CRMP2 (**a**) and CRMP4 (**c**); semi-quantitative Western blotting for CRMP2 (**b**) and CRMP4 (**d**). Overall, the CRMP2 expression increased with age and was not different in BD animals as compared to age-matched normal controls. The level of CRMP4 in normal controls decreased towards month 4 and again increased at the age of 6 months, and was higher in all BD animals as compared to age-matched normal controls. Asterisks above bars denote significant differences (*P* < 0.05) for a given region between age groups of a given condition (normal or BD). Numbers above BD-related bars denote the % statistical difference between BD and age-matched normal control groups (*P* < 0.05). Results are means with ± SD. Abbreviations: C – central, P – peripheral

2-D DIGE revealed a diverse effect of age and BD on the different CRMP2 isoforms (Fig. 3a). Some isoforms increase, others decrease with age in normal but not in BD kittens. When probing for the overall CRMP2 expression level by Western analysis low levels were detected in 1N and 2N groups, which increased to the adult level by the age of 4 months (Fig. 3b). This age-dependent increase in CRMP2 expression was also observed in early onset BD animals but lasted longer, until 6 months of age (Fig. 3a, b). Nevertheless, in comparison to age-matched normal controls the total amount of CRMP2 did not differ for either early or late onset BD kittens (Fig. 3a, b).

In contrast to CRMP2, all CRMP4 isoforms showed a similar age dependent profile in normal and BD kittens as visualized by 2-D DIGE (Fig. 3c). Western blotting also revealed this initial decrease in expression until month 4, and an increase towards the age of 6 months, to achieve adult levels (Fig. 3d). In all BD conditions CRMP4 levels were higher as compared to age-matched normal controls in both regions of area 17 (Fig. 3c, d; Table 2). Nevertheless the expression profile remains parallel to the normal developmental course.

**Table 2:**
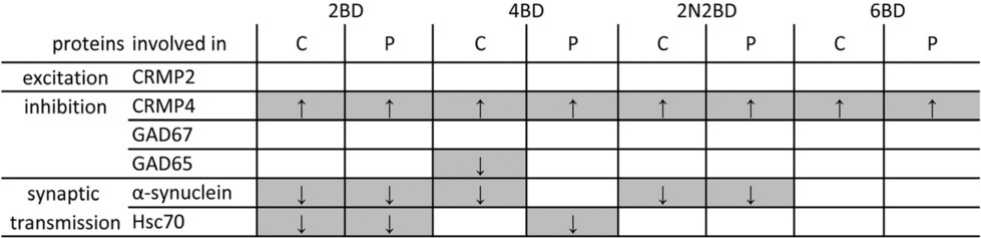
Results summary

Summary of protein expression differences between BD animals and their age-matched normal controls as investigated by semi-quantitative Western blotting. The highest number of protein expression changes is observed in 2BD and the lowest in 6BD kittens. See Fig. 2a to follow interactions between analyzed proteins. Arrows denote difference and its direction as compared to age-matched normal control groups. Abbreviations: C – central region, P – peripheral region, white squares – lack of difference between BD animals and age-matched normal controls

#### Synaptic transmission

**Fig. 4:**
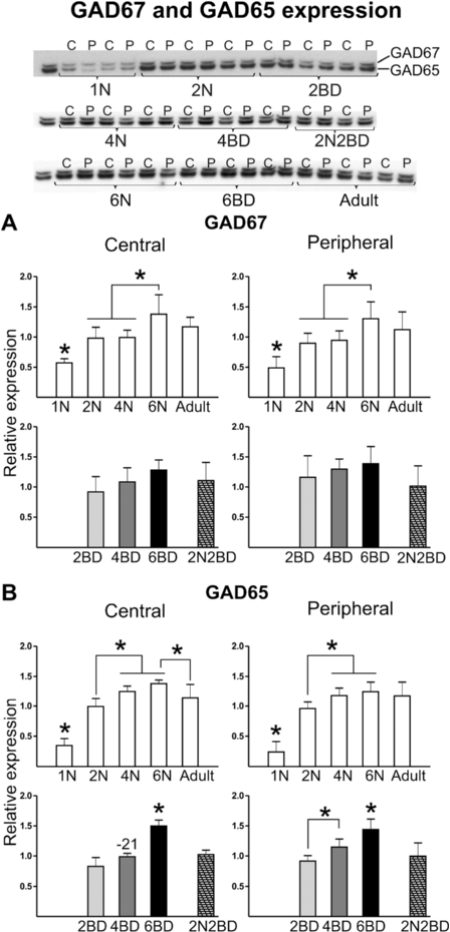
Semi-quantitative Western blotting for proteins involved in GABA synthesis: GAD67 (**a**) and GAD65 (**b**). GAD67 expression increased with age and was not different in BD animals as compared to age-matched normal controls. Nevertheless, the increase between month 4 and 6 observed in BD animals was not present in normal animals. GAD65 expression also increased with age, with a significantly slower rate in BD as compared to normal animals in both regions of area 17, achieving the highest level at 6 instead of 4 months. Additionally, in the central region of 4BD kittens GAD65 did not show a developmental increase, maintaining a lower level as compared to the 4N group. Together all observations are indicative for a delay in the development of normal cortical inhibition levels. Asterisks above bars denote significant differences (*P* < 0.05) for a given region between age groups of a given condition (normal or BD). Numbers above BD-related bars denote the % statistical difference between BD and age-matched normal control groups (*P* < 0.05). Results are means with ± SD

GAD67 produces the main cellular pool of GABA, whereas GAD65 produces GABA that is preferentially packed into vesicles for fast neurotransmitter use [22–29]. The expression of GAD65 and GAD67 increased during normal cortical development achieving mature levels at 6 months of age for GAD67 and at 4 months for GAD65 for both central and peripheral area 17 (Fig. 4a, b). In early and late onset BD animals GAD67 expression levels did not differ from age-matched normal controls or across regions (Fig. 4a). The only difference we detected relates to the lack of a significant increase in GAD67 levels between month 4 and 6. The developmental increase of GAD65 expression was slower in BD compared to normal animals in both regions of area 17, achieving the highest level only in 6BD kittens (Fig. 4b). Additionally, in the central region of 4BD kittens GAD65 did not show a developmental increase, maintaining a lower level as compared to the 4N group (Fig. 4b). As such, the GAD65 expression level in 4BD was similar to that in 2N kittens (Fig. 4b). In 2N2BD, GAD65 expression did not differ from age-matched 4N controls or younger 2N animals. However, similar to 4BD, 2N2BD showed a lower GAD65 level than 6BD in both regions, indicating that both late onset and short early onset BD arrests the developmental increase in GAD65 levels (Fig. 4b).

**Fig. 5:**
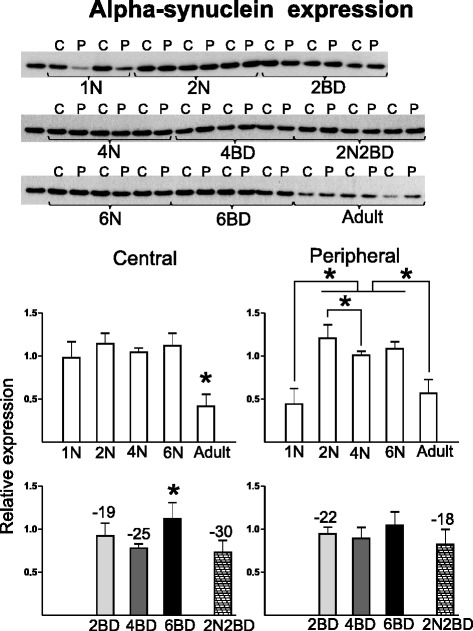
Semi-quantitative Western blotting for alpha-synuclein. Alpha-synuclein had a lower level in both area 17 regions of 2BD kittens and in the central region of the 4BD group as compared to age-matched normal controls. Additionally, a higher level of alpha-synuclein was observed in the central as compared to the peripheral region in 1N kittens. Asterisks above bars denote significant differences (*P* < 0.05) for a given region between age groups of a given condition (normal or BD). Numbers above BD-related bars denote the % statistical difference between BD and age-matched normal control groups (*P* < 0.05). Results are means with ± SD

Alpha-synuclein is involved in the regulation of vesicle storage and turnover as well as in the maturation and modulation of synaptic function [30–32]. It promotes SNARE-complex assembly during exocytosis in presynaptic terminals [32]. The Western blot experiment showed that specifically at the age of 1 month alpha-synuclein expression is lower in peripheral area 17. This centro-peripheral gradient is lost from 2N onwards. After the age of 6N, both cortical regions exhibit a drop to adult expression levels (Fig. 5). Western analysis confirmed the 2-D DIGE result showing a lower level of alpha-synuclein in the central region of 2BD kittens as compared to age-matched normal controls (Fig. 5). In 2N2BD subjects both central and peripheral area 17 showed a lower alpha-synuclein level as compared to age-matched normal controls, exhibiting a similar effect to that observed for early onset, age-matched 4BD kittens (Fig. 5).

#### Clathrin-mediated endocytosis

**Fig. 6:**
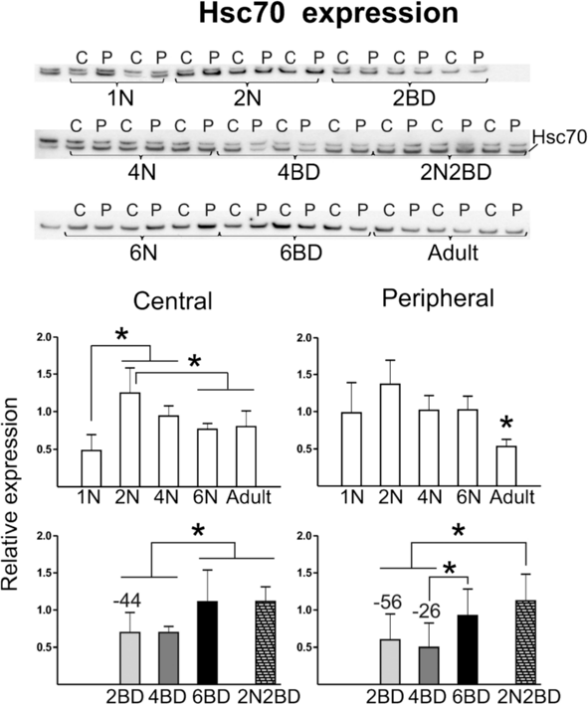
Semi-quantitative Western blotting for Hsc70. Hsc70 expression in BD animals showed lower levels in both regions of 2BD and also in the peripheral region of the 4BD group as compared to age-matched normal controls. Contrary to 4BD animals, Hsc70 expression in the 2N2BD group did not differ as compared to age-matched 4N group regions. Additionally, a lower level of Hsc70 was observed in central as compared to peripheral region in 1N kittens. Asterisks above bars denote significant differences (*P* < 0.05) for a given region between age groups of a given condition (normal or BD). Numbers above BD-related bars denote the % statistical difference between BD and age-matched normal control groups (*P* < 0.05). Results are means with ± SD

Western analysis for Hsc70 revealed an opposite expression profile for early onset BD kittens compared with normal animals (Fig. 6). In normal kittens, a decrease from the age of 2 months into adulthood characterized both regions of area 17, and in the 1 N group Hsc70 expression was lower in central as compared to peripheral area 17 (Fig. 6). However, in early onset BD animals expression was lower and only reached normal levels by 6BD (Fig. 6). The effects of late onset 2N2BD differed markedly from those observed in early onset age-matched 4BD animals, but not from age-matched normal controls. Together, this could suggest that Hsc70 plays an important role in early development since it is mostly affected by a lack of pattern vision from eye opening.

## Discussion

Normal development of primary visual cortex is shaped by visual experience and mirrored by region-specific activity reporter gene expression, in conjunction with the previously described central-to-peripheral maturation gradients in the visual system (reviewed in [11]). During normal development, in cat, the peripheral region of area 17 still undergoes intensive developmental changes between the 2^nd^ and 4^th^ month of age when its central counterpart is already in a more mature state [8]. We also witnessed such a centro-peripheral maturation gradient in 1N and 2N kittens based on protein expression patterns. As predicted, BD exerted a cortical region-specific effect on these protein expression profiles. Several of these developmental protein expression changes occurring between 2 and 4 months in normal animals (Table 1; column normal/age; rows 1–20) were absent in BD subjects. In fact, protein expression in peripheral area 17 hardly differed between 4BD kittens and the younger 2N animals, confirming our previous results where we showed that BD exerts a stronger delay effect on the maturation of peripheral area 17 as measured by visually-induced activity reporter gene *zif268* expression [8]. Additionally, under BD, centro-peripheral differences were observed up to 4 months. Together, these observations indicate that BD may not only delay but also prolong and enhance the centro-peripheral protein expression gradient related to the development of area 17. In sum, by exploiting the impact of BD on cortical maturation we could implicate four biological processes and thirty-six proteins in visual cortex development.

### Metabolism

The constant energy demand for protein synthesis needed for neurite outgrowth and the formation of new connections was reflected by changes in protein expression related to energy metabolism. It has been suggested before that processes that underlie synaptic plasticity may rely on changes in energy metabolism and expression of genes and proteins involved in metabolic processes, which is often defined as ‘metabolic plasticity’ [33–37] (reviewed in [38]). According to our 2-D DIGE data, 2 months of BD from birth, but not 4 months, induces a wide range of changes in expression of proteins involved in energy production, indicating the presence of a period of enhanced visual cortex plasticity. 2BD animals may thus exhibit more plasticity potential as compared to 2N animals, which are indeed already in the declining phase of the critical period for ocular dominance plasticity [39–41]. Indeed, in 1N kittens expression of mitochondrial genes was higher [42]. This hypothesis is in line with recent work [8], where we showed that 2BD kittens exhibited a pattern of high molecular activity in area 17, similar to 1-month-old controls, which are at the peak of the critical period for ocular dominance plasticity [40, 43]. Dark rearing also induces an upregulation of several mitochondrial genes (ATPase 6, cytochrome b, NADH dehydrogenase subunit 4 and 2; [42]). Altogether, these observations indicate that the high expression of proteins involved in energy metabolism may not only reflect the neuronal activity level and metabolic demand, but also is an indicator of neuronal plasticity. The upregulation of L-lactate dehydrogenase in 2BD kittens supports this interpretation, as L-lactate signaling was shown to be involved in plasticity processes during memory formation where it mediates molecular changes such as induction of phospho-CREB, Arc and phospho-cofilin [44]. Furthermore, L-lactate derived from astrocytes also affects NMDA receptor signaling and induces expression of IEGs including *arc*, *zif268* and *c-fo*s *in vitro* and *in vivo*, inducers of synaptic plasticity and activity [45].

### Outgrowth

The differential regulation of CRMP2 and CRMP4 under BD may be related to their cell-type specific expression and to the signaling pathways related to structural plasticity they are involved in. CRMP2 mediates repulsive semaphorin3A (Sema3A) signaling through the Rac-dependent pathway [46, 47] or a Rho kinase-dependent cascade reaction. Growth cone collapse is induced by activation of RhoA GTPase and Rho kinase (ROCK), leading to subsequent microtubule disassembly (reviewed in [48]). CRMP4 also interacts with RhoA GTPase [49], but is not known to be a ROCK substrate. While both CRMP2 and CRMP4 are capable of binding to tubulin heterodimers [50], CRMP4 is suggested to interact mainly with the actin cytoskeleton as it was shown to bundle with F-actin [51]. These data suggest that BD may reorganize the actin cytoskeleton in cat area 17 in a way that slows down the developmentally regulated formation of new connections.

We observed an age-dependent decrease in CRMP4 expression in both regions of area 17 as previously observed for cats and rodents [1, 2, 52, 53]. Crucial to the interpretation of our data, CRMP2 and CRMP4 exhibit cell-type specific expression. CRMP4-immunoreactive neurons appear mainly as non-pyramidal and parvalbumin-positive, a marker for a distinct subset of inhibitory interneurons, whereas CRMP2-immunopositive neurons are mainly parvalbumin-negative and display a clear pyramidal shape, typical for excitatory neurons [3, 54]. The development of inhibitory connectivity in cat area 17, measured by the number of symmetric synapses, follows a linear increase that reaches adult values by month 4 [55, 56], when the lowest CRMP4 expression is observed. A correlation between the CRMP4 expression profile and the development of inhibitory connectivity would be in line with the specific immuno-localization of CRMP4 in parvalbumin-positive interneurons [3].

### Synaptic transmission

Consistent with previous results by Guo and coworkers [5], in animals with normal visual experience, GAD65 and GAD67 achieved adult expression levels at 4 and 6 months of age respectively, in line with observations in humans [57]. In BD animals we observed a delay in the developmental increase of GAD65. In mice with a reduced synthesis of GABA, due to knockout of the gene encoding GAD65, ocular dominance plasticity is not present and the critical period is postponed until an appropriate level of inhibition is experimentally acquired [58, 59]. Experience-dependent plasticity therefore requires a minimal level of cortical inhibition to establish a threshold in the excitation/inhibition balance (for review see [60, 61]). The delay in the developmental increase in GAD65 in BD kittens thus reflects the delayed maturation of area 17. Dark rearing was shown to decrease the number of GAD65 puncta on layer 2/3 pyramidal neurons in mouse visual cortex [62, 63]. In rat, immature GABAergic inhibition in the visual cortex has also been observed in the context of dark-rearing from birth [64]. Together these observations indicate that visual experience is permissive to reach mature inhibitory networks. In the 2N2BD group no difference in GAD expression was detected when compared to age-matched normal controls. It is not surprising since a late-onset dark rearing period, preceded by normal visual experience during the first 3 weeks of life, also did not change GABAergic transmission in 5-week-old dark reared rats [64].

The expression of alpha-synuclein, a modulator of synaptic neurotransmission, was higher in kittens than in adult cats. Interestingly, for alpha-synuclein we observed higher levels in central as compared to peripheral area 17 in 1-month-old normal control kittens. A Zebra Finch homolog of alpha-synuclein is expressed in the song control nucleus when song plasticity is at its maximum [65]. Thus, the higher expression of alpha-synuclein in the central region at 1N may be an indicator of an earlier critical period onset for central area 17 as compared to peripheral area 17 [66]. Alpha-synuclein was shown to promote exocytosis. Its decreased expression in both regions of 2BD and in the central region of 4BD kittens, in combination with our observations for GAD65 and CRMP4, may suggest a negative regulation of inhibitory neurotransmission [30, 32].

### Clathrin-mediated endocytosis

In our study a higher level of Hsc70 is observed in kittens than in adult cats, which is in line with a higher intensity of receptor recycling in younger animals [67]. High levels of Hsc70 may be related to an increased intensity of clathrin-mediated endocytosis responsible for developmental rearrangements in N-methyl-D-aspartate receptor (NMDAR) subunit (NR1, NR2A, NR2B) number and composition at the excitatory glutamate synapse (Fig. 2a, [68]). In kittens, all NMDAR subunits show a peak expression at 2 months of age with a subsequent decline into adulthood [7]. Much of the decline in expression of NMDAR subunits occurs between the age of 2 and 4 months in cat [6, 7]. Enhanced internalization of NMDARs at early developmental stages contributes to the preferential insertion of NR2B over NR2A, while the decline in NMDAR internalization during neuronal maturation may be related to the stabilization of the mature NR2A/NR2B expression ratio at the synapse [68, 69] (reviewed in [70]).

BD resulted in a downregulation of Hsc70 in both area 17 regions in 2BD and in the peripheral region only of 4BD as compared to age-matched normal controls, in agreement with our previous observation that retinal lesion-induced visual cortex plasticity involves downregulation of Hsc70 [37]. Our 2-D DIGE data showed that the other protein indicated by IPA to be involved in clathrin-mediated endocytosis, endophilin-B2 [71], was also downregulated under BD, in the central region of 2BD kittens. Another protein that was shown to enhance clathrin-mediated endocytosis after neuronal stimulation is alpha-synuclein [72], which reveals a comparable modulation by BD to that observed for Hsc70.

Hsc70 may be involved in the formation of a complex of proteins that anchor GAD65 to the synaptic vesicle membrane [25, 29]. Thus, the decrease of Hsc70 expression up to the age of 4 months under early onset BD may not only indicate a downregulation of clathrin-mediated endocytosis but also a decrease in the activity of synaptic vesicles, reduced GABA synthesis and neurotransmission. Clathrin-mediated endocytosis is a major pathway for synaptic vesicle recycling (reviewed in [73]), thus downregulation of this pathway is again in line with downregulation of inhibitory synaptic transmission.

### Distinct behavioral outcomes of pattern deprivation in adult cats can be foreseen in the developmental molecular profiles

Overall, our assessment of the developmental cortical proteome confirms the recently described immature state of the primary visual cortex due to early onset BD [8]. For 2BD kittens we detected an upregulation of proteins involved in energy production as if the cortex is in a more immature state. 4BD kittens exhibited a delay in maturation specifically for peripheral area 17. Exactly the peripheral region of 4BD subjects had a proteomic signature of more immature 2-month-old normal control kittens and correspondingly, centro-peripheral protein expression gradients were detected up to 4 months of BD. This molecular immaturity of the primary visual cortex at the end of a given BD period seems to permit a different behavioral outcome in relation to the functional compensation induced by ensuing normal visual input [16]. Once adult, 2BD cats indeed outscore normal cats on most of the tested motion perception tasks. 4BD cats perform at the same level as normal subjects. In contrast, delayed onset 2N2BD or 6 BD cats show specific deficiencies in motion perception tasks [16], here mirrored by an adult-like molecular profile at the end of the BD period (summarized in Table 2), as described for *zif268* expression [8]. Specifically these two BD regimes did not induce a downregulation of Hsc70 (Table 2), a protein involved in clathrin-mediated endocytosis. 6BD subjects also did not show a downregulation of alpha-synuclein, a protein involved in synaptic transmission (Table 2) and show the most profound anatomical deficiencies in adulthood specific to motion perception, e.g., anatomical rearrangements of motion sensitive alpha retinal ganglion cells [14] and severe impairment of the simplest motion detection task [17 compare with 16, 18]. Most likely the impaired development of primary visual cortex becomes stabilized at some point, thereby preventing further modifications even in de context of restoration of normal visual experience.

## Conclusions

Taking advantage of the recently described impact of a postnatal BD period on primary visual cortex maturation [8] we could implicate four biological processes and thirty-six proteins in subregion-specific cortical development within area 17. We could correlate transient negative regulation of neurite outgrowth, synaptic transmission and clathrin-mediated endocytosis to the previously reported differences in behavioral outcome in adult cats with regard to the timing and duration of such a BD period in early life [16]. Combined, our findings suggest that the cortical plasticity potential to functionally recover from an early BD period once normal visual input is restored may evolve as a function of the specific sets of protein expression changes or delays, instigated by the loss of pattern vision in early life.

## Methods

### Animals

All experiments were carried out in accordance with the European Parliament and the Council Directive of September 22th 2010 (2010/63/EU). The cats were raised under a daily photoperiod of 12 h light and 12 h darkness with water and food *ad libitum* (Nencki Institute, Warsaw, Poland). All efforts were made to minimize animal discomfort. The cat brain material for this protein investigation has already been used in parallel in another study [8].

To screen for proteomic changes in relation to area 17 maturation under normal visual stimulation (N) and to screen for the effect of binocular deprivation from patterned visual experience (BD), we analyzed central and peripheral regions of area 17 of cats with normal visual experience at the age of two (2N, n = 3) and four (4N, n = 3) months and of cats binocularly deprived for either two (2BD, n = 3) or four (4BD, n = 3) months from eye opening (P8). Western blot analysis was also performed on additional animal groups: normal kittens of one (1N, n = 2) and six months (6N, n = 3), normal adult cats of 1–2 years (n = 3), kittens binocularly deprived for six months from eye opening (6BD, n = 3) and kittens from a delayed onset BD group that were deprived for the third and fourth month of life after two initial months of normal visual input (2N2BD, n = 3).

BD was always achieved by having the cats wearing double thickness linen masks covering their eyes. This procedure reduces retinal illumination to a similar level as lid suturing, but is less traumatic [74]. The masks were replaced daily in a normally lit animal facility room where the kittens lived. The changing procedure lasted no longer than one minute per day for each cat, which is not sufficient to maintain normal vision [75, 76] and allowed constant adjustment of the size of the masks to the growing head. The masks were removed at the end of the deprivation period.

Prior to administration of an overdose of sodium pentobarbital (Nembutal, 60 mg/kg, i.p.) all animals were maintained overnight in total darkness followed by 1-h light stimulation. Brains were dissected, instantly frozen by immersion in dry ice cooled isopetane (Poch, Gliwice, Poland) and stored at −80 °C.

### Protein extraction

We collected tissue from the central and peripheral region of area 17 at Horsley-Clarke level posterior 6.0 -7.0. Using the *in situ* hybridization films for *zif268* [8] and the cat visual cortex map of [77] as a guide, we punched ~10 mm^2^ cortical tissue, containing all cortical layers (Fig. 1a), from 3–4 consecutive 200 μm-thick frontal sections for each experimental and normal control condition to obtain sufficient protein material.

For 2-D DIGE, brain tissue was transferred to 100 μl ice-cold lysis buffer, containing 7 M urea (Chem-Lab), 2 M thiourea (Fluka), 4 % w/v CHAPS (Applichem), 1 % w/v dithiothreitol (DTT) (Applichem), 40 mM Tris base (Sigma), and Complete Protease Inhibitor Cocktail (Roche Diagnostics). Brain tissue was homogenized on ice, briefly centrifuged at 13 000 rpm, sonicated, followed by a complete solubilization of the proteins for 1 h at RT. The proteins were sonicated again and centrifuged for 20 min at 13 000 rpm at 4 °C to precipitate cell debris. The supernatant was dialyzed against milli-Q water for 2 h to remove residual salt using a membrane with a 500-Da cut-off (Spectra/Por, Biotech, Omnilabo). Protein concentrations were determined according to the Qubit^™^ Quantitation Platform (Invitrogen) using a Qubit^™^ fluorometer (Invitrogen, Merelbeke, Belgium). Samples were kept at −80 °C.

For Western blotting, the collected brain tissue was homogenized in 100 μl lysis solution (2 % w/v sodium dodecyl sulfate (SDS) [Sigma- Aldrich], 50 mM Tris–HCl [Sigma-Aldrich], 10% glycerol [Acros Organics], pH 6.8) containing 4 μl protease inhibitor (Complete Protease Inhibitor Cocktail tablets; Roche Diagnostics). After mechanical homogenization by drill-driven pestles, all the samples were sonicated for 5 x 10 s, heated at 70 °C for 5 min, and centrifuged for 20 min at 13 000 rpm at 4 °C. The supernatant was isolated and the total protein concentration was determined as described above.

### Two-dimensional difference Gel electrophoresis

The fluorescent cyanine dyes, Cy2 (Cy2), propyl-Cy3 (Cy3), and methyl-Cy5 (Cy5) were in-house synthesized [1, 2] according to the method by Ünlü et al. [78]. All other chemicals were purchased from GE Healthcare, unless mentioned otherwise. Pre-cast Immobiline DryStrips (24 cm, pH 3–11 nonlinear) were rehydrated overnight in DeStreak Rehydratation Solution containing 0.5 % v/v immobilized pH gradient (IPG) buffer in a reswelling tray covered with paraffin oil (Merck). The next day 50 μg protein of each cat area 17 sample was randomly labeled with either propyl-Cy3 or methyl-Cy5. Equal fractions of all samples were pooled and 50 μg of this pool was labeled with Cy2 to serve as an internal standard. The minimal amount of dye that gave a maximum number of spots and the highest signal-to-noise ratio was set to approximately 200 pmol as described previously [1, 2]. The samples were incubated for 30 min on ice in the dark during the labeling process; subsequently, the reaction was terminated by addition of 1 μl lysine (10 mM; Merck) for 15 min. The Cy2-, Cy3-, and Cy5-labeled fractions were mixed together, and an equal volume of lysis solution was added. Isoelectric focusing (IEF) was performed on an Ettan IPGphor Cup Loading Manifold system according to manufacturer’s instructions. Actual run conditions were 300 V for 3 h, 600 V for 3 h, followed by a 6-h gradient to 1000 V, a 3-h gradient to 8000 V, and 8 h at 8000 V for a total of 50 kVh (at 50 μA/strip). After IEF, the strips were equilibrated twice for 15 min in equilibration buffer (6 M urea, 34.5 % v/v glycerol and 10 % w/v SDS in Tris–HCl buffer [1.5 M, pH 8.8]). DTT (1 % w/v) was added to the first equilibration step and 4.5 % w/v iodoacetamide (Sigma-Aldrich) to the second step. Electrophoresis of the IPG strips was done on 1.5-mm-thick SDS-polyacrylamide gels (12.5 % T; 2.6 % C) in the Ettan DALT twelve system for 30 min at 30 mA and 24 h at 13 °C at 15 mA/gel.

#### 2-D DIGE gel image analysis and statistics

Gels were scanned with the Ettan DIGE Imager (software 1.0; GE Healthcare) and generated gel image triplets (Cy2, Cy3, and Cy5) comprising the CyDye-labeled proteins. Quantitative analysis was carried out with the DeCyder 2D difference analysis software (Version 7.0; GE Healthcare). Spot detection and matching was performed automatically with the DeCyder Batch processor. The gel-to-gel matching was also checked manually followed by statistical analysis of protein abundance change between samples in the biological variation analysis (BVA) module embedded in the DeCyder Software [37, 79, 80]. Spots of interest differentially expressed at least in two comparisons with *p* < 0.05, and spots differentially expressed in one comparison with *p* < 0.01 were further analyzed with MS (39 spots fulfilled the criteria, Fig. 1b).

### Protein identification

Two preparative gels were run under the same conditions as described above with the exception of the first dimension separation, which was run for a total of 50 kVh (at 50 μA/strip). Each gel was loaded with 1 mg of protein from the pool sample, from which only a 50-μg fraction was labeled with Cy3. Glass plates were pretreated with BindSilane, and 2 reference markers were applied to enable automatic spot picking. The preparative gels were scanned in the Ettan DIGE Imager to obtain an image of the Cy3 signal.

Subsequently, the total protein load was visualized by Lava Purple total protein fluorescent stain according to manufacturer’s instructions (Fluorotechnics), and the gels were scanned again. Matching with the analytical gels was as before carried out automatically with manual correction by the BVA module of the DeCyder software. A pick list of the proteins of interest was generated and imported into the Spot Picker Version 1.20 software that controls the Ettan Spot Picker (GE Healthcare).

### Protein identification

In collaboration with the Centre de Recherche Public-Gabriel Lippmann in Luxemburg, MS and MS/MS spectra were acquired using a 5800 MALDI TOF-TOF (Absciex, Sunnyvale, CA, U.S.A.) and calibrated using the 4700 peptide mass calibration kit (Applied Biosystems). Proteins were identified by searching against the NCBI database, limited to the taxonomy Mammalia (downloaded on 4/06/2012, 1,063,527 sequences), using an in-house MASCOT server (version 2.3.0 Matrix Science, www.matrixscience.com, London, U.K.). All searches were carried out defining trypsin as cleavage agent and allowing for 2 missed cleavages. A mass window of 100 ppm was tolerated for the precursor mass and 0.75 Da for fragment ion masses. The search parameters allowed for carboxymethylation of cysteine as fixed modification and oxidation of methionine and tryptophan (double oxidation, and kynurenin formation) as variable modifications. Proteins were considered as identified when two, none overlapping, individual peptides surpassed the peptide score threshold or when the expect value <5.0e-005. When this criterion was not met, additional precursors were selected and searched using the above-described parameters. In total, we identified 36 unique proteins (Additional file 1: Table S1) in 32 spots (all 39 spots classified for identification with MS are indicated in Fig. 1b). Occasionally, proteins with the same name were detected in more than one spot due to the fact that the 2-D DIGE method is sensitive enough to separate different isoforms and posttranslational modifications of one protein (Table 1, e.g., rows 2 and 3; CRMP2 and CRMP4). In one case, Western blot results were opposite to the 2-D DIGE findings, likely due to the co-occurrence of two proteins, Hsc70 and CRMP2, in one spot (nr 623). Therefore only the Western blot data were used to describe the Hsc70 results.

The 36 identified proteins from the 2-D DIGE analysis were analyzed by means of the QIAGEN’s Ingenuity Pathway Analysis (IPA®, QIAGEN Redwood City, www.qiagen.com/ingenuity) software. IPA calculates the significance value of a given canonical pathway as the probability that the pathway is associated with the data set by random chance. To reveal key biological pathways, functions and molecular networks comprising the identified proteins we applied the stringent Benjamin-Hochberg (B-H) multiple testing correction method.

### Western blotting and statistics

To obtain the optimal protein load, a protein dilution series was performed with total protein amounts of 0.5-25 μg per lane. A concentration that resulted in a good signal to noise ratio and still was in the linear range of the detection system was chosen. For CRMP2, CRMP4, Hsc70, alpha-synuclein, GAD65/67 this resulted in 2.5, 8, 4, 30 and 15 μg respectively. After the addition of 5 μl reducing agent (10x, Invitrogen) and 2 μl LDS sample buffer (4x, Invitrogen), the samples were denatured (10 min, 70 °C). Protein samples were loaded onto a 4-12 % Bis-Tris NuPagel gel (Invitrogen). Electrophoresis was carried out using the Xcell4 SureLock Midi-Cell module (Invitrogen) according to the manufacturer’s instructions and subsequently transferred to a PVDF or Nitrocellulose (for alpha-synuclein) membrane (iBlot, Gel Transfer Stack; Invitrogen). The Spectra^™^ Multicolor High range protein ladder (ThermoScientific) was used as molecular weight standard. After blotting, the membrane was incubated for 1–2 h in a 5% ECL blocking agent (GE Healthcare, Buckinghamshire, UK) in Tris-saline (0.01 M Tris, 0,9% NaCL, 0,1% TX-100, pH 7.6) and incubated overnight with a primary antibody (diluted in 5% blocking agent in Tris Saline) against CRMP2 (1/24000, mouse Ab, generously provided by Dr. Y. Ihara, University of Tokyo, Department of Neuropathology, Japan), CRMP4 (1/10000, rabbit Ab, AB5454 Millipore), Hsc70 (1/2000, rabbit Ab, SPA-816, Stressgen Bioreagents), alpha-synuclein (1/1000, rabbit Ab, in house produced and characterized by Prof. Veerle Baekelandt, KU Leuven, Department of Neurosciences, Belgium [81]), GAD65/67 (1/6000, rabbit Ab, ab11070, Abcam). The next day, the blots were washed in Tris-Saline (3x5min) and 30 min incubated with a horseradish peroxidase-conjugated secondary goat anti-mouse (GaM-HRP, 1/50000, Dako, Glostrup, Denmark), goat anti-rabbit (GaR-HRP, 1/50000; Dako, Glostrup, Denmark) or donkey anti-goat Ab (1/50000, sc-2020, Santa Cruz Biotechnology) (diluted in 5% blocking agent in Tris Saline), followed by a rinse in Tris-Saline (3x5min) and Tris-stock (1x5min; 0,05 M Tris, pH 7.6). Immunoreactivity was visualized using chemiluminescence detection (Supersignal West Dura, Thermo Scientific, Pierce) on ECL hyperfilm (GE Healthcare). The protein bands were semi-quantitatively evaluated by densitometry (ImageQuant TL v. 7.0; GE Healthcare). For the Hsc70 antibody two bands were detected in samples of young cats (up to 4 months), independent of visual manipulation, as previously reported for adult cats with retinal lesions [37]. Only the lower one (MW of 73 k kDa, [82]) was considered to be specific according to the molecular-weight size marker.

To account for intra-gel and inter-gel variability including loading differences, incomplete transfer, or position on the blot, a total protein stain (LavaPurple, Gelcompany) was used rather than the use of a single reference protein [37, 83] (Additional file 2: Figure S1, Additional file 3: Figure S2, Additional file 4: Figure S3, Additional file 5: Figure S4) according to manufacturer’s instructions. For semi-quantitative densitometry, non-uniform staining was corrected for by inter-lane measurements and normalization. For each protein of interest, the specific protein band per cat was normalized to its corresponding normalized total protein stain. Also, each experiment on the same cat was repeated two times. A reference sample (pool) consisting of a mixture of each prepared tissue sample was run with the same optimal amount of protein on each gel to gauge blot-to-blot variability (first left lane in each blot in Figs. 3, 4, 5 and 6). Statistical analysis of WB data was performed using a nested-design ANOVA model to investigate the effects of group, cortical region and blot nested in cat [84] by means of data analysis software system STATISTICA version 10. There was a significant effect of interaction between group and region for GAD65 (*p* = 0.0269, F = 2.48, df = 8), alpha-synuclein (*p* = 0.0001, F = 14.01, df = 8), Hsc70 (*p* = 0.0001, F = 6.305, df = 8). There was a significant effect of group for CRMP2 (*p* = 0.0056, F = 3.57, df = 6), CRMP4 (*p* = 0.0001, F = 68.60, df = 6) and GAD67 (*p* = 0.0001, F = 8.45, df = 6). A *post hoc* test was carried out using the Tukey HSD method. Statistical differences were indicated for *p* < 0.05.

## Additional files

Additional file 1: Table S1. List of proteins identified by mass spectrometry. (PDF 104 kb) Additional file 2: Figure S1. Total protein stain for normalization of CRMPs specific bands. (PDF 607 kb) Additional file 3: Figure S2. Total protein stain for normalization of GADs specific bands. (PDF 331 kb) Additional file 4: Figure S3. Total protein stain for normalization of α-synuclein specific bands. (PDF 316 kb) Additional file 5: Figure S4. Total protein stain for normalization of Hsc70 specific bands. (PDF 262 kb)

## Abbreviations

2-D: Two-Dimensional
BD: binocular deprivation
C: central
c: clathrin-mediated endocytosis
CRMP: collapsin response mediator protein
DIGE: Difference Gel Electrophoresis
GAD: Glutamic acid decarboxylase
Hsc70: Heat shock cognate 70
IEF: Isoelectric focusing
IPA: Ingenuity Pathway Analysis
LGN: lateral geniculate nucleus
m: energy metabolism
mR: mRNA metabolism and transport
MS: mass spectometry
N: normal
NMDA: N-methyl-D-aspartate receptor
o: outgrowth
P: peripheral
ROCK: RhoA GTPase and Rho kinase
s: synaptic transmission
WB: Western blot

## References

[1] Van den Bergh G, Clerens S, Firestein BL, Burnat K, Arckens L (2006). Development and plasticity-related changes in protein expression patterns in cat visual cortex: A fluorescent two-dimensional difference gel electrophoresis approach. Proteomics.

[2] Van den Bergh G, Clerens S, Cnops L, Vandesande F, Arckens L (2003). Fluorescent two-dimensional difference gel electrophoresis and mass spectrometry identify age-related protein expression differences for the primary visual cortex of kitten and adult cat. J Neurochem.

[3] Cnops L, Hu T-T, Burnat K, Van der Gucht E, Arckens L (2006). Age-dependent alterations in CRMP2 and CRMP4 protein expression profiles in cat visual cortex. Brain Res.

[4] Cnops L, Hu T-T, Burnat K, Arckens L (2008). Influence of Binocular Competition on the Expression Profiles of CRMP2, CRMP4, Dyn I, and Syt I in Developing Cat Visual Cortex. Cereb Cortex.

[5] Guo Y, Kaplan IV, Cooper NG, Mower GD (1997). Expression of two forms of glutamic acid decarboxylase (GAD67 and GAD65) during postnatal development of cat visual cortex. Brain Res Dev Brain Res.

[6] Mower GD, Chen L (2003). Laminar distribution of NMDA receptor subunit (NR1, NR2A, NR2B) expression during the critical period in cat visual cortex. Mol Brain Res.

[7] Chen L, Cooper NGF, Mower GD (2000). Developmental changes in the expression of NMDA receptor subunits (NR1, NR2A, NR2B) in the cat visual cortex and the effects of dark rearing. Mol Brain Res.

[8] Laskowska-Macios K, Zapasnik M, Hu T-T, Kossut M, Arckens L, Burnat K. Zif268 mRNA Expression Patterns Reveal a Distinct Impact of Early Pattern Vision Deprivation on the Development of Primary Visual Cortical Areas in the Cat. Cereb Cortex. 2014;doi:10.1093/cercor/bhu192 [Epub ahead of print]. 10.1093/cercor/bhu192 PMC4585500 25205660

[9] Hata Y, Ohshima M, Ichisaka S, Wakita M, Fukuda M, Tsumoto T (2000). Brain-derived neurotrophic factor expands ocular dominance columns in visual cortex in monocularly deprived and nondeprived kittens but does not in adult cats. J Neurosci.

[10] Schmidt KE, Stephan M, Singer W, Löwel S (2002). Spatial analysis of ocular dominance patterns in monocularly deprived cats. Cereb Cortex.

[11] Burnat K (2015). Are Visual Peripheries Forever Young?. Neural Plast.

[12] O'Kusky JR (1985). Synapse elimination in the developing visual cortex: a morphometric analysis in normal and dark-reared cats. Brain Res.

[13] Bourne JA, Warner CE, Rosa MG (2005). Topographic and laminar maturation of striate cortex in early postnatal marmoset monkeys, as revealed by neurofilament immunohistochemistry. Cereb Cortex.

[14] Burnat K, Van der Gucht E, Waleszczyk WJ, Kossut M, Arckens L (2012). Lack of early pattern stimulation prevents normal development of the alpha (Y) retinal ganglion cell population in the cat. J Comp Neurol.

[15] Sherman SM, Spear PD (1982). Organization of visual pathways in normal and visually deprived cats. Physiol Rev.

[16] Zapasnik M, Burnat K (2013). Binocular pattern deprivation with delayed onset has impact on motion perception in adulthood. Neuroscience.

[17] Burnat K, Vandenbussche E, Zernicki B (2002). Global motion detection is impaired in cats deprived early of pattern vision. Behav Brain Res.

[18] Burnat K, Stiers P, Arckens L, Vandenbussche E, Zernicki B (2005). Global form perception in cats early deprived of pattern vision. NeuroReport.

[19] Ellemberg D, Lewis TL, Maurer D, Brar S, Brent HP (2002). Better perception of global motion after monocular than after binocular deprivation. Vision Res.

[20] Lewis TL, Ellemberg D, Maurer D, Wilkinson F, Wilson HR, Dirks M, Brent HP (2002). Sensitivity to global form in glass patterns after early visual deprivation in humans. Vision Res.

[21] Bento-Abreu A, Velasco A, Polo-Hernández E, Lillo C, Kozyraki R, Tabernero A, Medina JM (2009). Albumin endocytosis via megalin in astrocytes is caveola- and Dab-1 dependent and is required for the synthesis of the neurotrophic factor oleic acid. J Neurochem.

[22] Erlander MG, Tillakaratne NJ, Feldblum S, Patel N, Tobin AJ (1991). Two genes encode distinct glutamate decarboxylases. Neuron.

[23] Feldblum S, Erlander MG, Tobin AJ (1993). Different distributions of GAD65 and GAD67 mRNAs suggest that the two glutamate decarboxylases play distinctive functional roles. J Neurosci Res.

[24] Solimena M, Aggujaro D, Muntzel C, Dirkx R, Butler M, De Camilli P, Hayday A (1993). Association of GAD-65, but not of GAD-67, with the Golgi complex of transfected Chinese hamster ovary cells mediated by the N-terminal region. Proc Natl Acad Sci U S A.

[25] Hsu CC, Davis KM, Jin H, Foos T, Floor E, Chen W, Tyburski JB, Yang CY, Schloss JV, Wu JY (2000). Association of L-glutamic acid decarboxylase to the 70-kDa heat shock protein as a potential anchoring mechanism to synaptic vesicles. J Biol Chem.

[26] Wu H, Jin Y, Buddhala C, Osterhaus G, Cohen E, Jin H, Wei J, Davis K, Obata K, Wu J-Y (2007). Role of glutamate decarboxylase (GAD) isoform, GAD65, in GABA synthesis and transport into synaptic vesicles—Evidence from GAD65-knockout mice studies. Brain Res.

[27] Kanaani J, Patterson G, Schaufele F, Lippincott-Schwartz J, Baekkeskov S (2008). A palmitoylation cycle dynamically regulates partitioning of the GABA-synthesizing enzyme GAD65 between ER-Golgi and post-Golgi membranes. J Cell Sci.

[28] Kaufman DL, Houser CR, Tobin AJ (1991). Two forms of the gamma-aminobutyric acid synthetic enzyme glutamate decarboxylase have distinct intraneuronal distributions and cofactor interactions. J Neurochem.

[29] Jin H, Wu H, Osterhaus G, Wei J, Davis K, Sha D, Floor E, Hsu CC, Kopke RD, Wu JY (2003). Demonstration of functional coupling between γ-aminobutyric acid (GABA) synthesis and vesicular GABA transport into synaptic vesicles. Proc Natl Acad Sci U S A.

[30] Cabin DE, Shimazu K, Murphy D, Cole NB, Gottschalk W, McIlwain KL, Orrison B, Chen A, Ellis CE, Paylor R, Lu B, Nussbaum RL (2002). Synaptic vesicle depletion correlates with attenuated synaptic responses to prolonged repetitive stimulation in mice lacking alpha-synuclein. J Neurosci.

[31] Murphy DD, Rueter SM, Trojanowski JQ, Lee VM (2000). Synucleins are developmentally expressed, and alpha-synuclein regulates the size of the presynaptic vesicular pool in primary hippocampal neurons. J Neurosci.

[32] Burre J, Sharma M, Tsetsenis T, Buchman V, Etherton MR, Sudhof TC (2010). Alpha-synuclein promotes SNARE-complex assembly in vivo and in vitro. Science.

[33] Nie F, Wong-Riley MT (1996). Mitochondrial- and nuclear-encoded subunits of cytochrome oxidase in neurons: differences in compartmental distribution, correlation with enzyme activity, and regulation by neuronal activity. J Comp Neurol.

[34] Lachance PED, Chaudhuri A (2004). Microarray analysis of developmental plasticity in monkey primary visual cortex. J Neurochem.

[35] Magistretti PJ (2011). Neuron–glia metabolic coupling and plasticity. Exp Physiol.

[36] Ding Q, Vaynman S, Souda P, Whitelegge JP, Gomez-Pinilla F (2006). Exercise affects energy metabolism and neural plasticity-related proteins in the hippocampus as revealed by proteomic analysis. Eur J Neurosci.

[37] Hu T-T, Van den Bergh G, Thorrez L, Heylen K, Eysel UT, Arckens L (2011). Recovery from Retinal Lesions: Molecular Plasticity Mechanisms in Visual Cortex Far beyond the Deprived Zone. Cereb Cortex.

[38] Magistretti PJ (2006). Neuron-glia metabolic coupling and plasticity. J Exp Biol.

[39] Hubel DH, Wiesel TN (1970). The period of susceptibility to the physiological effects of unilateral eye closure in kittens. J Physiol.

[40] Cynader M, Mitchell DE (1980). Prolonged sensitivity to monocular deprivation in dark-reared cats. J Neurophysiol.

[41] Daw NW, Gordon B, Fox KD, Flavin HJ, Kirsch JD, Beaver CJ, Ji Q, Reid SN, Czepita D (1999). Injection of MK-801 affects ocular dominance shifts more than visual activity. J Neurophysiol.

[42] Yang C, Silver B, Ellis SR, Mower GD (2001). Bidirectional regulation of mitochondrial gene expression during developmental neuroplasticity of visual cortex. Biochem Biophys Res Commun.

[43] Daw NW, Fox K, Sato H, Czepita D (1992). Critical period for monocular deprivation in the cat visual cortex. J Neurophysiol.

[44] Suzuki A, Stern SA, Bozdagi O, Huntley GW, Walker RH, Magistretti PJ, Alberini CM (2011). Astrocyte-neuron lactate transport is required for long-term memory formation. Cell.

[45] Yang J, Ruchti E, Petit J-M, Jourdain P, Grenningloh G, Allaman I, Magistretti PJ (2014). Lactate promotes plasticity gene expression by potentiating NMDA signaling in neurons. Proc Natl Acad Sci U S A.

[46] Yamashita N, Morita A, Uchida Y, Nakamura F, Usui H, Ohshima T, Taniguchi M, Honnorat J, Thomasset N, Takei K, Takahashi T, Kolattukudy P, Goshima Y (2007). Regulation of spine development by semaphorin3A through cyclin-dependent kinase 5 phosphorylation of collapsin response mediator protein 1. J Neurosci.

[47] Brown M, Jacobs T, Eickholt B, Ferrari G, Teo M, Monfries C, Qi RZ, Leung T, Lim L, Hall C (2004). Alpha2-chimaerin, cyclin-dependent Kinase 5/p35, and its target collapsin response mediator protein-2 are essential components in semaphorin 3A-induced growth-cone collapse. J Neurosci.

[48] Charrier E, Reibel S, Rogemond V, Aguera M, Thomasset N, Honnorat J (2003). Collapsin response mediator proteins (CRMPs): involvement in nervous system development and adult neurodegenerative disorders. Mol Neurobiol.

[49] Alabed YZ, Pool M, Tone SO, Fournier AE (2007). Identification of CRMP4 as a Convergent Regulator of Axon Outgrowth Inhibition. J Neurosci.

[50] Fukata Y, Itoh TJ, Kimura T, Menager C, Nishimura T, Shiromizu T, Watanabe H, Inagaki N, Iwamatsu A, Hotani H, Kaibuchi K (2002). CRMP-2 binds to tubulin heterodimers to promote microtubule assembly. Nat Cell Biol.

[51] Rosslenbroich V, Dai L, Baader SL, Noegel AA, Gieselmann V, Kappler J (2005). Collapsin response mediator protein-4 regulates F-actin bundling. Exp Cell Res.

[52] Cnops L, Van de Plas B, Arckens L (2004). Age-dependent expression of collapsin response mediator proteins (CRMPs) in cat visual cortex. Eur J Neurosci.

[53] Tsutiya A, Ohtani-Kaneko R (2012). Postnatal alteration of collapsin response mediator protein 4 mRNA expression in the mouse brain. J Anat.

[54] Demeulemeester H, Arckens L, Vandesande F, Orban GA, Heizmann CW, Pochet R (1991). Calcium binding proteins and neuropeptides as molecular markers of GABAergic interneurons in the cat visual cortex. Exp Brain Res.

[55] Winfield DA (1983). The postnatal development of synapses in the different laminae of the visual cortex in the normal kitten and in kittens with eyelid suture. Brain Res.

[56] Winfield DA (1981). The postnatal development of synapses in the visual cortex of the cat and the effects of eyelid closure. Brain Res.

[57] Pinto JG (2010). Developmental changes in GABAergic mechanisms in human visual cortex across the lifespan. Front Cell Neurosci.

[58] Hensch TK, Fagiolini M, Mataga N, Stryker MP, Baekkeskov S, Kash SF (1998). Local GABA circuit control of experience-dependent plasticity in developing visual cortex. Science.

[59] Fagiolini M, Hensch TK (2000). Inhibitory threshold for critical-period activation in primary visual cortex. Nature.

[60] Jiang B, Huang ZJ, Morales B, Kirkwood A (2005). Maturation of GABAergic transmission and the timing of plasticity in visual cortex. Brain Res Rev.

[61] Sale A, Berardi N, Spolidoro M, Baroncelli L, Maffei L (2010). GABAergic inhibition in visual cortical plasticity. Front Cell Neurosci.

[62] Chattopadhyaya B, Di Cristo G, Higashiyama H, Knott GW, Kuhlman SJ, Welker E, Huang ZJ (2004). Experience and activity-dependent maturation of perisomatic GABAergic innervation in primary visual cortex during a postnatal critical period. J Neurosci.

[63] Kreczko A, Goel A, Song L, Lee H-K (2009). Visual Deprivation Decreases Somatic GAD65 Puncta Number on Layer 2/3 Pyramidal Neurons in Mouse Visual Cortex. Neural Plast.

[64] Morales B, Choi S-Y, Kirkwood A (2002). Dark rearing alters the development of GABAergic transmission in visual cortex. J Neurosci.

[65] George JM, Jin H, Woods WS, Clayton DF (1995). Characterization of a novel protein regulated during the critical period for song learning in the zebra finch. Neuron.

[66] Rathjen S, Schmidt KE, Löwel S (2003). Postnatal growth and column spacing in cat primary visual cortex. Exp Brain Res.

[67] Philpot BD, Sekhar AK, Shouval HZ, Bear MF (2001). Visual Experience and Deprivation Bidirectionally Modify the Composition and Function of NMDA Receptors in Visual Cortex. Neuron.

[68] Roche KW, Standley S, McCallum J, Dune Ly C, Ehlers MD, Wenthold RJ (2001). Molecular determinants of NMDA receptor internalization. Nat Neurosci.

[69] Lavezzari G, McCallum J, Dewey CM, Roche KW (2004). Subunit-specific regulation of NMDA receptor endocytosis. J Neurosci.

[70] Nong Y, Huang Y-Q, Salter MW (2004). NMDA receptors are movin’ in. Curr Opin Neurobiol.

[71] Irie F, Okuno M, Pasquale EB, Yamaguchi Y (2005). EphrinB-EphB signalling regulates clathrin-mediated endocytosis through tyrosine phosphorylation of synaptojanin 1. Nat Cell Biol.

[72] Gedalya BT, Loeb V, Israeli E, Altschuler Y, Selkoe DJ, Sharon R (2009). Alpha-synuclein and polyunsaturated fatty acids promote clathrin-mediated endocytosis and synaptic vesicle recycling. Traffic.

[73] Dittman J, Ryan TA (2009). Molecular circuitry of endocytosis at nerve terminals. Annu Rev Cell Dev Biol.

[74] Kossut M, Michalski A, Zernicki B (1978). The ocular following reflex in cats deprived of pattern vision from birth. Brain Res.

[75] Schwarzkopf DS, Vorobyov V, Mitchell DE, Sengpiel F (2007). Brief daily binocular vision prevents monocular deprivation effects in visual cortex. Eur J Neurosci.

[76] Mitchell DE, Sengpiel F, Hamilton DC, Schwarzkopf DS, Kennie J. Protection against deprivation amblyopia depends on relative not absolute daily binocular exposure. J Vis. 2011;11. 10.1167/11.7.13 21680647

[77] Rosenquist AC, Jones APEG (1985). Connections of visual cortical areas in the cat. Cerebral Cortex.

[78] Ünlü M, Morgan ME, Minden JS (1997). Difference gel electrophoresis: a single gel method for detecting changes in protein extracts. Electrophoresis.

[79] Willems E, Hu T-T, Soler Vasco L, Buyse J, Decuypere E, Arckens L, Everaert N (2014). Embryonic Protein Undernutrition by Albumen Removal Programs the Hepatic Amino Acid and Glucose Metabolism during the Perinatal Period in an Avian Model. PLoS ONE.

[80] Van Hove I, Verslegers M, Hu T-T, Carden M, Arckens L, Moons L. A proteomic approach to understand MMP-3-driven developmental processes in the postnatal cerebellum: Chaperonin CCT6A and MAP kinase as contributing factors. Devel Neurobio. 2015;doi: 10.1002/dneu.22272 [Epub ahead of print] 10.1002/dneu.22272 25652596

[81] Van der Perren A, Toelen J, Casteels C, Macchi F, Van Rompuy A-S, Sarre S, Casadei N, Nuber S, Himmelreich U, Osorio Garcia MI, Michotte Y, D'Hooge R, Bormans G, Van Laere K, Gijsbers R, Van den Haute C, Debyser Z, Baekelandt V (2015). Longitudinal follow-up and characterization of a robust rat model for Parkinson’s disease based on overexpression of alpha-synuclein with adeno-associated viral vectors. Neurobiol Aging.

[82] Doong H, Rizzo K, Fang S, Kulpa V, Weissman AM, Kohn EC (2003). CAIR-1/BAG-3 abrogates heat shock protein-70 chaperone complex-mediated protein degradation. J Biol Chem.

[83] Aldridge GM, Podrebarac DM, Greenough WT, Weiler IJ (2008). The use of total protein stains as loading controls: an alternative to high-abundance single-protein controls in semi-quantitative immunoblotting. Proc Natl Acad Sci U S A.

[84] Pavlidis P (2003). Using ANOVA for gene selection from microarray studies of the nervous system. Methods.

[85] Soghomonian JJ, Martin DL (1998). Two isoforms of glutamate decarboxylase: why?. Trends Pharmacol Sci.

[86] Beites CL, Xie H, Bowser R (1999). The septin CDCrel-1 binds syntaxin and inhibits exocytosis. Proc Natl Acad Sci U S A.

[87] Kinoshita A, Noda M, Kinoshita M (2000). Differential localization of septins in the mouse brain. J Comp Neurol.

[88] Borisoff JF, Chan CC, Hiebert GW, Oschipok L, Robertson GS, Zamboni R, Steeves JD, Tetzlaff W (2003). Suppression of Rho-kinase activity promotes axonal growth on inhibitory CNS substrates. Mol Cell Neurosci.

[89] Fredrich M, Illing RB (2010). MMP-2 is involved in synaptic remodeling after cochlear lesion. NeuroReport.

[90] Eustace BK, Jay DG (2004). Extracellular roles for the molecular chaperone, hsp90. Cell Cycle.

[91] Liu J, Zhang J-P, Shi M, Quinn T, Bradner J, Beyer R, Chen S, Zhang J (2009). Rab11a and HSP90 regulate recycling of extracellular alpha-synuclein. J Neurosci.

[92] Sundborger A, Soderblom C, Vorontsova O, Evergren E, Hinshaw JE, Shupliakov O (2011). An endophilin-dynamin complex promotes budding of clathrin-coated vesicles during synaptic vesicle recycling. J Cell Sci.

[93] Newmyer SL, Schmid SL (2001). Dominant-interfering Hsc70 mutants disrupt multiple stages of the clathrin-coated vesicle cycle in vivo. J Cell Biol.

[94] Krecic AM (1999). Swanson MS: hnRNP complexes: composition, structure, and function. Curr Opin Cell Biol.

[95] Coyle JT, Enna SJ (1976). Neurochemical aspects of the ontogenesis of GABAergic neurons in the rat brain. Brain Res.

[96] Perez-Olle R, Lopez-Toledano MA, Goryunov D, Cabrera-Poch N, Stefanis L, Brown K, Liem RKH (2005). Mutations in the neurofilament light gene linked to Charcot-Marie-Tooth disease cause defects in transport. J Neurochem.

[97] Menazza S, Wong R, Nguyen T, Wang G, Gucek M, Murphy E (2013). CypD(−/−) hearts have altered levels of proteins involved in Krebs cycle, branch chain amino acid degradation and pyruvate metabolism. J Mol Cell Cardiol.

[98] Ishizaki H, Miyoshi J, Kamiya H, Togawa A, Tanaka M, Sasaki T, Endo K, Mizoguchi A, Ozawa S, Takai Y (2000). Role of rab GDP dissociation inhibitor alpha in regulating plasticity of hippocampal neurotransmission. Proc Natl Acad Sci U S A.

[99] Takai Y, Sasaki T, Shirataki H, Nakanishi H (1996). Rab3A small GTP-binding protein in Ca^(2+)^-dependent exocytosis. Genes Cells.

[100] Mukherjee J, DeSouza LV, Micallef J, Karim Z, Croul S, Siu KWM, Guha A (2009). Loss of collapsin response mediator Protein1, as detected by iTRAQ analysis, promotes invasion of human gliomas expressing mutant EGFRvIII. Cancer Res.

[101] Letourneur F, Gaynor EC, Hennecke S, Demolliere C, Duden R, Emr SD, Riezman H, Cosson P (1994). Coatomer is essential for retrieval of dilysine-tagged proteins to the endoplasmic reticulum. Cell.

[102] Arimura N, Inagaki N, Chihara K, Menager C, Nakamura N, Amano M, Iwamatsu A, Goshima Y, Kaibuchi K (2000). Phosphorylation of collapsin response mediator protein-2 by Rho-kinase. Evidence for two separate signaling pathways for growth cone collapse. J Biol Chem.

[103] Waterman-Storer CM, Karki SB, Kuznetsov SA, Tabb JS, Weiss DG, Langford GM, Holzbaur EL (1997). The interaction between cytoplasmic dynein and dynactin is required for fast axonal transport. Proc Natl Acad Sci U S A.

[104] Rodriguez-Rodriguez RA, Tabernero A, Velasco A, Lavado EM, Medina JM (2004). The neurotrophic effect of oleic acid includes dendritic differentiation and the expression of the neuronal basic helix-loop-helix transcription factor NeuroD2. J Neurochem.

[105] Tabernero A, Lavado EM, Granda B, Velasco A, Medina JM (2008). Neuronal differentiation is triggered by oleic acid synthesized and released by astrocytes. J Neurochem.

