# Supplementary material for: Binocular pattern deprivation interferes with the expression of proteins involved in primary visual cortex maturation in the cat
Source: Mol Brain. 2015 Aug 14;8:48. doi: 10.1186/s13041-015-0137-7 (PMC4536594; doi:10.1186/s13041-015-0137-7)
Supplement: Additional file 1: Table S1. — List of proteins identified by mass spectrometry. (PDF 104 kb) [file 13041_2015_137_MOESM1_ESM.pdf]

SUPPLEMENTARY TABLE 1

Spot number	Protein name	NCBI protein accession number	Mowse score	Type of difference	Average ratio	p-value
389	heat shock protein HSP 90-alpha isoform 2	154146191	1230	4BDC>4NC	1.39	0.00093
				4BDC>4BDP	1.18	0.0038
				4BDC>2BDC	1.76	0.005
513	albumin	30962111	934	4BDC>4NC	2.73	0.016
				4NC<2NC	2.35	0.0072
537	collapsin response mediator protein 2	348587934	338	2NP>2NC	1.61	0.034
				2NP>4NP	1.44	0.045
	heat shock 70 kDa protein 12A	301764321	113	4BDP>4NP	1.23	0.017
567	NFL	116283387	725	2BDC>2NC	2.11	0.01
				4BDC>2BDC	1.75	0.0062
623	collapsin response mediator protein 2	40254595	513	4BDC>4NC	1.52	0.048
				4BDC>2BDC	1.68	0.045
	heat shock cognate 71 kDa protein (Hsc70)	42542422	158	4NP>2NP	1.33	0.041
628	collapsin response mediator protein 2	40254595	735	4BDC>2BDC	1.26	0.016
				4NC>2NC	1.78	0.041
717	heterogeneous nuclear ribonucleoprotein L	348563003	294	2BDC>2NC	2.02	0.019
				2BDC>4BDC	2.60	0.00053
				4BDC<4BDP	1.51	0.034
				4BDC<4NC	1.38	0.039
737	rab GDP dissociation inhibitor alpha<br>tubulin alpha-6 chain	71534276	516	2NC>4NC	1.12	0.011
				2NP>4NP	1.15	0.047
762	collapsin response mediator protein 1	300795989	639	2BDC>4BDC	2.06	0.003
				2NP>4NP	1.94	0.04
764	collapsin response mediator protein 4	301780602	381	2BDC>4BDC	1.58	0.047
				2NP>4NP	1.62	0.0089
765	collapsin response mediator protein 4<br>MAGUK p55 subfamily member 2	148678070<br>7710062	101<br>80	2NP>4NP	1.59	0.0029
769	collapsin response mediator protein 4	301780602	419	2BDC>4BDC	1.67	0.0063
	alpha-1-syntrophin	126723593	138	2BDC>2NC	1.57	0.023
	MAGUK p55 subfamily member 2	7710062	77	2NP>4NP	2.12	0.0046
772	collapsin response mediator protein 4	148678070	94	2BDP>2NP	1.53	0.049
				2BDC>4BDC	1.54	0.048
				2NP>4NP	1.86	0.0093
773	collapsin response mediator protein 4	148678070	96	2BDC>2NC	1.43	0.049
				2BDC>4BDC	1.66	0.01
				2BDP>4BDP	1.62	0.0062
				2NC>4NC	1.31	0.0085
				2NP>4NP	1.82	0.0077
774	collapsin response mediator protein 4	301780602	442	2NP>4NP	2.12	0.0098
889	glucose-6-phosphate isomerase<br>ATP synthase subunit alpha, mitochondrial	301779638<br>296222590	197<br>133	2BDP>2NP	1.31	0.005
913	glutamate dehydrogenase 1	148692928	750	2BDP>2BDC	1.15	0.0074
				2BDP>2NP	1.34	0.00044
974	ATP synthase subunit beta, mitochondrial	73968432	520	2BDC<2NC	1.68	0.035
	endophilin-B2<br>dynactin subunit 2	21314838<br>51948450	221<br>123	4NP>2NP	2.45	0.029
1162	peptidyl-prolyl cis-trans isomerase D (cyclophilin D)	13385854	110	2BDC>2NC	1.44	0.0054

1222	septin 5	149019803	446	2NP>2NC	1.52	0.033
				4NC>2NC	1.38	0.027
1351	Isocitrate dehydrogenase [NAD] subunit alpha, mitochondrial	344247643	284	2NP>2NC	1.42	0.023
				4NC>2NC	1.48	0.023
1577	coatomer subunit epsilon isoform 4	345787561	201	2NC>4NC	1.58	0.0052
1816	methylglutaconyl-CoA hydratase, mitochondrial	73946980	228	2NP>2NC	1.60	0.00092
				4NC>2NC	1.93	0.003
				4BDP>4NP	1.67	0.027
1930	peroxiredoxin-6	348577965	584	2NC>2NP	1.42	0.0082
				4NC>2NC	1.18	0.0075
				4NP>2NP	1.48	0.022
2126	glutathione S-transferase P	301771848	450	4NC>2NC	1.47	0.016
				4BDP>2BDP	1.42	0.04
2189	ATP synthase subunit d, mitochondrial	149723319	210	4NC>2NC	1.32	0.038
				4BDP>2BDP	1.39	0.029
2422	beta-synuclein	348575007	292	2NC>2NP	1.87	0.0086
2508	alpha-synuclein	355720963	341	2BDC<2NC	1.75	0.033
				4BDP>2BDP	2.15	0.034
2835	cytochrome b-c1 complex subunit 7	348588407	223	2BDC>2NC	2.01	0.018
				4BDC>4NC	1.98	0.046
	cytochrome c	351705793	128	2BDC>2BDP	1.38	0.049
2854	hemoglobin subunit alpha	122405	272	4BDC>4BDP	1.57	0.016
				4BDC>4NC	2.18	0.0027
	hemoglobin subunit beta	237823749	469	4BDP>4NP	1.70	0.014
2943	L-lactate dehydrogenase B chain	74136507	214	2BDP>2NP	1.61	0.02
				2BDP>4BDP	2.03	0.025
2951	heterogeneous nuclear ribonucleoprotein H<br>aldehyde dehydrogenase, mitochondrial	10946928<br>345790871	464<br>130	2BDP>2NP	1.43	0.0023

Supplemental Table 1. List of proteins identified by mass spectrometry that show significant changes in expression, ordered according to spot number, with the molecular weight of a protein increasing with spot number. The following types of comparisons were analyzed: 2BD and 4BD group vs their age-matched controls, 2N vs 4N and 2BD vs 4BD for each region separately, as well as central vs peripheral within each group. Columns represent: protein name, NCBI protein excession no., Mowse score, expression ratio, p value. Abbreviations: BD – binocular pattern deprivation, N – normal; C – central region, P – peripheral region.
