# Supplementary figures and images for: Binocular pattern deprivation interferes with the expression of proteins involved in primary visual cortex maturation in the cat
Source: Mol Brain. 2015 Aug 14;8:48. doi: 10.1186/s13041-015-0137-7 (PMC4536594; doi:10.1186/s13041-015-0137-7)

## CRMP2 expression

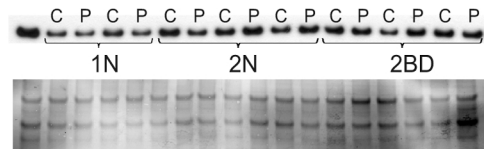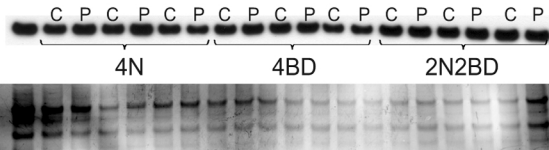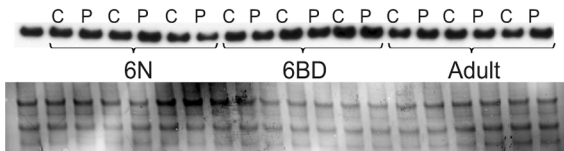

## CRMP4 expression

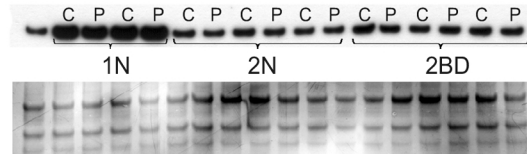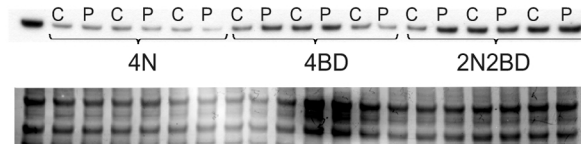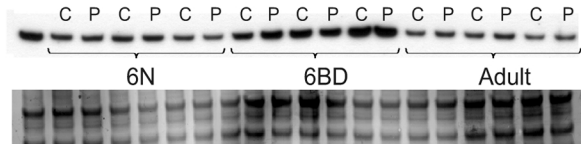

Supplement: Additional file 2: Figure S1. — Total protein stain for normalization of CRMPs specific bands. (PDF 607 kb) [file 13041_2015_137_MOESM2_ESM.pdf]

# GAD67 and GAD65 expression

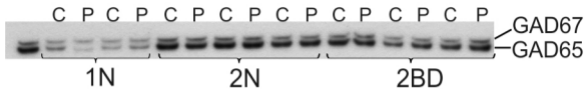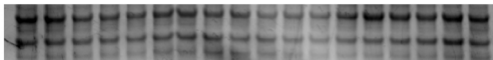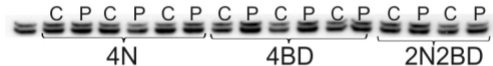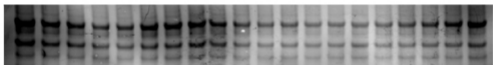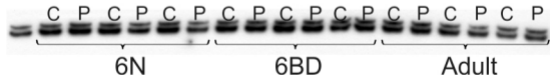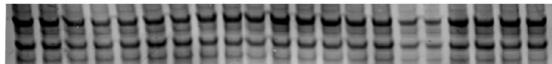

Supplement: Additional file 3: Figure S2. — Total protein stain for normalization of GADs specific bands. (PDF 331 kb) [file 13041_2015_137_MOESM3_ESM.pdf]

# Alpha-synuclein expression

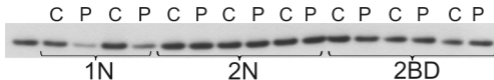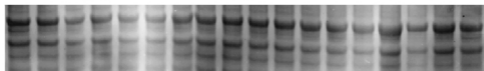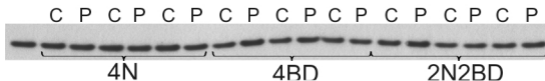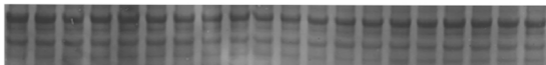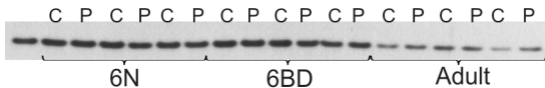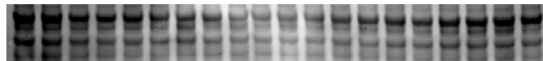

Supplement: Additional file 4: Figure S3. — Total protein stain for normalization of α-synuclein specific bands. (PDF 316 kb) [file 13041_2015_137_MOESM4_ESM.pdf]

# Hsc70 expression

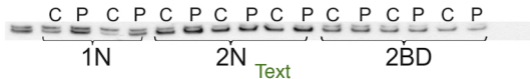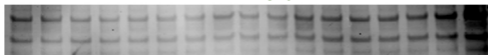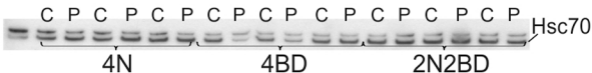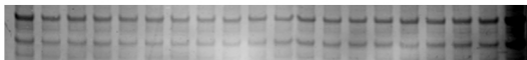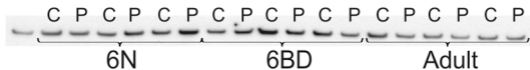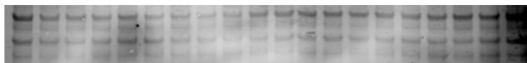

Supplement: Additional file 5: Figure S4. — Total protein stain for normalization of Hsc70 specific bands. (PDF 262 kb) [file 13041_2015_137_MOESM5_ESM.pdf]
